# The key characteristics concept

**DOI:** 10.1016/j.cotox.2024.100515

**Published:** 2025-03

**Authors:** Martyn T. Smith

**Affiliations:** School of Public Health, University of California, Berkeley, CA 94720, USA

**Keywords:** Carcinogens, Hazard, Risk assessment, Toxicity, AOPs, Cancer

## Abstract

In evaluating whether a chemical can cause cancer or another adverse outcome, three lines of evidence are typically considered: epidemiology, animal bioassays and mechanistic evidence. The key characteristics (KCs) form the basis of a uniform approach for searching, organizing, and evaluating mechanistic evidence to support hazard identification. KCs are the established properties of the toxicants themselves and are generated from our understanding of mechanisms of toxicity. KCs have been published for carcinogens, endocrine disruptors and reproductive, liver immune and cardiovascular toxicants. We noted that several KCs were common to different types of toxicants, whereas others were highly specific. Hence, there may be overlapping umbrella KCs for potentially hazardous bioactive chemicals that could be used in predictive toxicology. There are, however, also clearly unique KCs for chemicals that primarily target a specific organ and these unique KCs could be especially important to predicting target organ toxicity. It is possible that *in silico* approaches, *in vitro* tests, and *in vivo* biomarkers could be developed, which predict the “umbrella” and “unique” KCs of hazardous chemicals. However, given the significance of human evidence, the development of a set of biomarkers that could be used to measure the KCs in molecular epidemiology studies is also important.

## Introduction

In evaluating whether a chemical can cause cancer or another adverse outcome, three lines of evidence are typically considered: findings from epidemiological studies, animal bioassays and mechanistic evidence. The number of epidemiological studies is usually quite limited and methods for their evaluation, including meta-analysis, are well established. Similarly, pathologists and statisticians are able to effectively evaluate animal bioassays using existing methods. These are also typically low in number and are becoming less frequently performed. On the other hand, mechanistic studies are increasing in number and the literature is often voluminous encompassing hundreds if not thousands of studies [1,2].

Recognizing the above, in 2012, the International Agency for Research on Cancer (IARC) considered how mechanistic evidence could best be evaluated at two meetings organized as part of their review of the 100 IARC monographs produced to date [3]. The 10 key characteristics (KCs) of carcinogens arose as a consensus from these meetings and were eventually published in 2016 in a slightly different order from the original set [2,4]. The KCs are the inherent properties of the carcinogenic agents themselves and apply to chemical, physical, and biological agents. They address the major mechanisms through which cancer can be induced [5]. A critical point is that these KCs are agnostic toward any particular adverse outcome pathway since nearly all human carcinogens affect multiple pathways and endpoints [6]. The KCs can be used to systematically evaluate the peer-reviewed scientific literature to establish the biological plausibility for an adverse effect occurring in humans. Under the framework that is typically used, the strength of the mechanistic evidence for each KC is determined and can be used, if needed, to develop a probable mode of action. This evaluation involves expert review of the mechanistic studies that have been performed in humans, animals, and microorganisms or through new approach methods and takes into account the study quality and the scientific value of the toxicological and molecular epidemiological endpoints measured. Once this KC evaluation is complete, it is then used within an evidence synthesis or triangulation approach that includes the human epidemiological evidence, the available experimental animal data on apical endpoints together with the mechanistic evidence evaluated through the KCs as described above. On their own, the KCs cannot establish that a substance is a carcinogen, nor are they a checklist that if the evidence for a certain number are found to be strong then the substance is carcinogenic. However, under the guidelines provided in the updated IARC preamble [7], if there is “*strong evidence that the agent exhibits key characteristics of carcinogens”*, then an agent may be listed as a *“possible human carcinogen* (Group 2 B).”

## Adoption and expansion of the KC approach

The KC approach has been systematically employed in analyzing the mechanistic literature across multiple volumes of IARC Monographs, beginning in 2015 as recently reviewed by Rusyn and Wright [8]. IARC have also recently reviewed their experience using the KCs of carcinogens in a workshop and the findings of that workshop will be published shortly. For genotoxic and mutagenic carcinogens, it is typical for them to be electrophilic or converted to an electrophile metabolite (KC1) and for them to be genotoxic (KC2). If the evidence for these KCs is strong, it does not really matter if the substance possesses other KCs as the induction of mutations is a clear mechanism of carcinogenicity. Recent examples of electrophilic/genotoxic agents classified by IARC using the KCs include acrolein [9] and acrylonitrile [10]. For non-genotoxic carcinogens, evidence for other KCs is often cited in their classification. Classic examples include dioxins and polychlorinated biphenyls that act on the Ah receptor under KC8. Additionally, more recently strong evidence for KC 4 (causes epigenetic alterations) and KC7 (is immunosuppressive) played a role in IARC’s classification of perfluoro-octanoic acid [11] and KC6 (induces chronic inflammation) in the classification of talc [10].

The KCs of carcinogens are now also used by the United States (U.S.) National Toxicology Program Report on Carcinogens (NTP-RoC) [12], the U.S. Environmental Protection Agency IRIS Program (EPA-IRIS), and California Environmental Protection Agency’s (CalEPA) Office of Environmental Health Hazard Assessment [1] to inform decisions about the likelihood that a chemical or other agent is a carcinogen under their guidelines. Others have used to KCs to identify potential breast carcinogens [13] and to evaluate the mechanistic data relevant to the potential carcinogenicity of per- and poly-fluorinated substances (PFAS) [14,15], acetaminophen [16], glyphosate [17], aspartame [18] and chromium [19], to name but a few. At present I am not aware of any European or Asian regulatory agency that has used the KCs in their evaluations.

Responding to guidance from the National Academies, KCs have also been developed and published for endocrine disruptors [20] and reproductive [21,22], liver [23], immune [24,25] and cardiovascular toxicants [26]. Publication of KCs of metabolism disruptors and neurotoxicants is expected soon. It may also be valuable to develop KCs for kidney and pulmonary toxicants.

**Figure 1:**
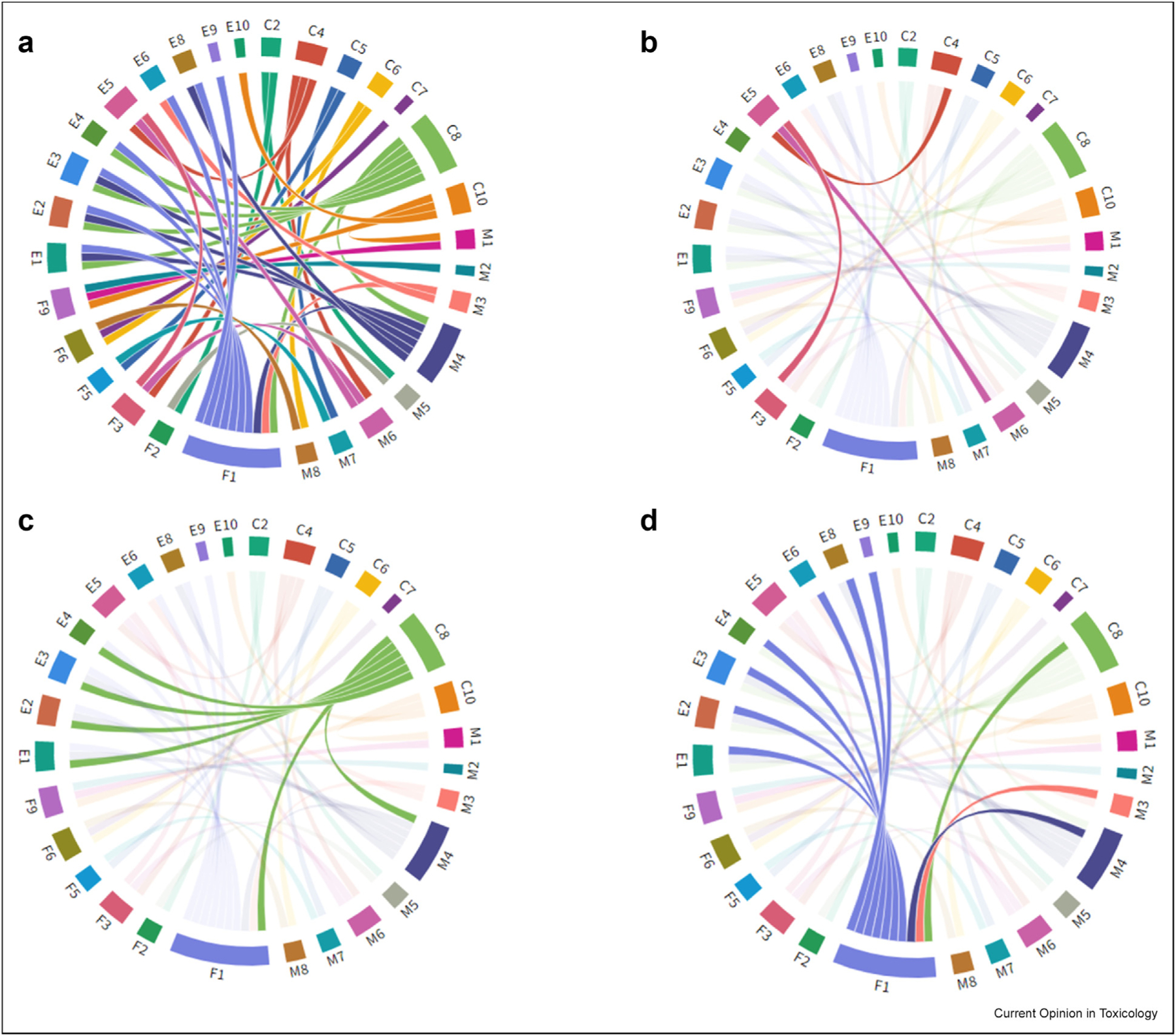
Chord diagram representing the interrelationships between key characteristics (KCs) of carcinogens (C), male reproductive toxicants (M), female reproductive toxicants (F), and endocrine disrupting chemicals (E). KCs for each group were mapped against each other (Fig.1a) indicating many overlaps and examples of overlapping KCs are shown for epigenetic alterations (Fig 1b), receptor-mediated effects (Fig.1c), and hormone signaling (Fig.1d).

Following the development of KCs for multiple forms of toxicity, we noted early on that several KCs were common to different types of toxicants, whereas others were highly specific (Figure 1). Hence, there may be “umbrella” KCs for potentially hazardous bioactive chemicals that could be used in predictive toxicology. There are; however, also clearly “unique” KCs for chemicals which primarily target a specific organ and these unique KCs could be especially important to predicting target organ toxicity. It is possible that *in silico* approaches, *in vitro* tests, and *in vivo* biomarkers could be developed which predict the “umbrella” and “unique” KCs of hazardous chemicals. However, given the importance of human evidence, the development of a set of biomarkers that could be used to measure the KCs and/or key events from endorsed adverse outcome pathways (AOPs) in molecular epidemiology studies is perhaps of paramount importance.

The ‘“umbrella” KCs appear to include the induction of oxidative stress, inflammation, epigenetic changes, and receptor-mediated effects. The latter also encompasses hormone-related signaling. The chord diagrams in Figure 1 show the interrelationships between the KCs of carcinogens (C), male reproductive toxicants (M), female reproductive toxicants (F), and endocrine disrupting chemicals (E). Many overlaps can be seen in Figure 1a and there are also multiple overlaps specifically for epigenetic alterations (Figure 1b), receptor-mediated effects (Figure 1c), and hormone signaling (Figure 1d). There has been some hesitancy on the part of committees formed to discuss future KC development to make too much of these overlapping umbrella effects as some members consideredcontext to be critical. For example, the role of oxidative stress in cancer development through the production of DNA damage may be quite different to its role in disrupting endocrine systems or causing neurotoxicity. Hence, if assays or NAMs are to be developed for umbrella KCs, it will be important to consider the organ or tissue context in which these effects occur. For example, the induction of oxidative stress in transformed cell lines may be largely irrelevant especially at doses approaching cytotoxicity.

Unique KCs can clearly be found for specific forms of toxicity, e.g. causes immortalization for carcinogens. Immune, liver and cardiovascular toxicants have several unique KCs that are quite different to the KCs of carcinogens. As mentioned above, these may be especially useful for predicting target organ toxicity and developing new approach methods (NAMs) that are contextually appropriate.

## Further development of the KC approach

### 1) Development of an appropriate set of assays, NAMs, and biomarkers

The significance of evidence in exposed humans for hazard identification is often forgotten in the era of the development of NAMs. It is also apparent that most current NAMs do not directly measure the KCs of carcinogens [27] and *in silico* approaches are lacking [28]. There is therefore the need, not only for the development of novel biomarkers but also NAMs that address each of the KCs or at the least measure a significant number of the key events that contribute to each KC.

The KCs of carcinogens were originally conceived to allow for the systematic retrospective analysis of the mechanistic literature, which can be voluminous for well-studied chemicals [29]. It is unclear if the KCs can be used prospectively to develop specific tests to measure them. The KCs are very broad categories, such as ‘modulates receptor-mediated effects’ or ‘induces epigenetic alterations’. Clearly many assays, NAMs, or biomarkers would be needed to assess such broad effects. Of interest is the fact that most of the KCs of carcinogens are related to so-called non-genotoxic events. Only one KC is specifically related to genotoxicity and the induction of mutations i.e. “Is genotoxic” [2]. There are already well-established methods for assessing genotoxicity and mutations in humans, experimental animals, and tissue culture. *In silico* analysis is also possible through various software programs, such as the OECD toolbox. The same is not true for non-genotoxic carcinogens or the key events that are involved in non-genotoxic carcinogenicity [30].

### 2) Integration of the KCs with adverse outcome pathways (AOPs)

The KCs of carcinogens and indeed the current state of the science on cancer development [31,32] clearly point to the need to develop methods that assess endpoints other than genotoxicity and mutagenicity. I would suggest that this is where the “adverse outcome pathway” (AOP) approach and the KC approach could merge. By categorizing the key events in the development to cancer from the relevant AOPs in relation to each of the 10 KCs of carcinogens, a systematic approach to the development of an appropriate set of assays, NAMs, and biomarkers could be developed.

The above is also true for other forms of toxicity. I would suggest that by using the individual KCs as a guide, AOPs could be used to assign relevant molecular-initiating events and key events to each KC. This would allow for the development of a set of NAMs, assays, and biomarkers that together would provide information of mechanistic relevance regarding the strength of the evidence for each KC in question.

### 3) Development of *in silico* approaches

Computational models that can predict the KCs of carcinogens and other forms of toxicity are also needed. In 2021, Tice et al. [28] evaluated the availability of *in silico* models for each KC of carcinogens. They identified numerous data gaps that hinder development of a *in silico* carcinogenicity protocol for regulatory use, which we are attempting to address. Computational methods exist to assess KC1 “is electrophilic” [33] and KC2 “is genotoxic” [34], but methods to assess the remaining KCs require development. Considerable progress has been made on KC8 “Modulates receptor-mediated effects” via our publicly available web server NR-ToxPred that predicts agonist and antagonist activity against more than 10 nuclear receptors (http://nr-toxpred.cchem.berkeley.edu/) [35]. Borrel and Rudel [36] have also developed a cheminformatics approach to identify structural features associated with estrogen or progesterone steroid production that helps address KC8. I am hopeful that improvements in AI and other advanced computing approaches may help to overcome many of the remaining challenges in predicting the other KCs of carcinogens *in silico*.

### 4) Application of artificial intelligence

Artificial intelligence (AI) and other computational approaches have the potential to advance application of the KCs of carcinogens in cancer hazard assessment. A database containing all methods used to evaluate each of the ten KCs in the IARC monographs and beyond has been developed. The purpose of this database is to provide a comprehensive mapping of published assays to each KC and to generate a roadmap for using information from these assays and models in evaluating the strength of the evidence for each KC. The database will soon be publicly available as a “living document” and will continue to be updated and refined. Moreover, AI using natural language processing can be employed to systematically gather, organize, and analyze all pertinent information related to each KC. Natural language processing enables the extraction of detailed data from diverse textual sources, including scientific articles, reports, and databases, which discuss the methods used to evaluate the carcinogenic properties of various chemicals. This approach may facilitate the automated categorization and summarization of relevant findings, thus enhancing the speed and robustness of the evidence synthesis process.

## Conclusion

The KCs form the basis of a uniform approach for searching, organizing, and evaluating mechanistic evidence to support hazard identification. They are becoming widely used by regulatory agencies and other authoritative bodies to evaluate mechanistic data. KCs are the established properties of the toxicants themselves and are generated from our understanding of mechanisms of toxicity. KCs have been published for carcinogens, endocrine disruptors and reproductive, liver, immune and cardiovascular toxicants. KCs for several other types of toxicants are in development.

It has been more than a decade since the original KCs of carcinogens were first formulated on November 30, 2012, at an IARC meeting in Lyon and they have been used by IARC Working Groups since 2015. From 2015 to 2022, a total of 19 IARC Monographs (73 agents combined) used KCs for cancer hazard classification [8]. Rusyn and Wright recently analyzed these monographs and found that mechanistic data from *in vivo* animal, *in vitro* animal, and *in vitro* human studies were most impactful in concluding that an agent could cause cancer via a particular KC [8]. They further found that to exclude the involvement of a KC, data from largescale systematic *in vitro* testing programs such as ToxCast, were most informative. They concluded that increased availability of systemized data streams, such as human *in vitro* data, would provide the basis for more confident and informed conclusions about both positive and negative associations and inform expert judgments on cancer hazard.

Here I have proposed that by using the individual KCs as a guide, AOPs could be used to assign relevant molecular-initiating events and key events to each KC. This would allow for the development of a set of NAMs, assays, and biomarkers that together would provide a systematized data stream to evaluate the strength of the evidence for each KC. I have further proposed that a set of NAMs, assays, and biomarkers be developed for both ‘umbrella’ and ‘unique’ KCs in a tissue/organ context that is appropriate to the type of toxicity being studied.

## Data Availability

No data was used for the research described in the article.
